# Machine learning models for adverse drug reaction prediction. A systematic review and three-level multilevel meta-analysis

**DOI:** 10.3389/fphar.2026.1906920

**Published:** 2026-09-14

**Authors:** Giorgio Cappello, Christoph Oster, Teresa Schmidt, Jana Grieger, Leon Jekel, Kathrin Kizina, Yahya Ahmadipour, Laurel Rauschenbach, Philipp Dammann, Ulrich Sure, Kathy Keyvani, Andreas Junker, Lazaros Lazaridis, Martin Stuschke, Christoph Pöttgen, Björn Scheffler, Christoph Kleinschnitz, Sied Kebir

**Affiliations:** 1 Department of Neurology and Center for Translational Neuro- and Behavioral Sciences (C-TNBS), Division of Clinical Neurooncology, University Medicine Essen, University Duisburg-Essen, Essen, Germany; 2 German Cancer Consortium (DKTK), Partner Site Essen/Dusseldorf, a Partnership Between DKFZ and University Hospital Essen, DKFZ-Division Translational Neurooncology at the West German Cancer Center (WTZ), University Medicine Essen, University Duisburg-Essen, Essen, Germany; 3 Department of Neurosurgery and Spine Surgery, University Medicine Essen, University Duisburg-Essen, Essen, Germany; 4 Institute of Neuropathology, University Medicine Essen, University Duisburg-Essen, Essen, Germany; 5 Department of Neurology, University Hospital Knappschaftskrankenhaus Bochum, Ruhr University Bochum, Bochum, Germany; 6 Department of Radiotherapy, University Medicine Essen, University Duisburg-Essen, Essen, Germany; 7 German Cancer Research Center (DKFZ), Heidelberg, Germany

**Keywords:** adverse drug reactions, clinical applicability, machine learning, meta-analysis, pharmacovigilance, PROBAST, systematic review

## Abstract

**Background:**

Adverse drug reactions (ADRs) are a leading cause of preventable hospitalization, yet the dominant pharmacovigilance paradigm remains reactive. Machine learning (ML) offers a data-driven alternative, but the evidence base has not been formally meta-analyzed under clinically realistic inclusion criteria and a dependence-respecting statistical framework. This is the first PRISMA 2020-compliant and PROBAST-screened multilevel meta-analysis of broad-spectrum ML-based ADR prediction. We aimed to quantify pooled discrimination of ML models for broad-spectrum ADR prediction and to test the influence of algorithm class, protein-target features, and outcome breadth.

**Methods:**

Following a Preferred Reporting Items for Systematic Reviews and Meta-Analyses (PRISMA) 2020 compliant, PROSPERO-registered protocol (CRD420250653686), we searched PubMed and Web of Science Core Collection from inception to 30 January 2025. A Scopus and IEEE Xplore top-up search on 2 February 2026 yielded no further records. Eligible studies applied ML to clinically validated data sources for multi-drug ADR prediction. Areas under the receiver operating characteristic curve (AUCs) were logit-transformed and pooled with a study-level DerSimonian-Laird random-effects model and a pre-specified primary three-level multilevel meta-regression (restricted maximum likelihood [REML]) using 30 model-level AUCs nested within 9 contributing studies.

**Results:**

Eleven Prediction model Risk Of Bias ASsessment Tool (PROBAST) screened studies were included, of which 9 contributed quantitatively. The pre-specified primary three-level multilevel pooled AUC was 0.841 (95% confidence interval [CI] 0.789-0.883), with a study-level DerSimonian-Laird sensitivity estimate of 0.832 (95% CI 0.788-0.869, I-squared 90.7%). Leave-one-study-out estimates ranged 0.818-0.842. Neural networks did not statistically outperform logistic regression, random forests, or k-nearest neighbor. An apparent protein-target feature penalty (model-level p = 0.0002) was driven by one dominant study and disappeared in multilevel sensitivity analysis (adjusted p = 0.75).

**Conclusion:**

ML models showed moderate discrimination under Grading of Recommendations Assessment, Development and Evaluation (GRADE) low certainty. Within this small and methodologically heterogeneous evidence base, the pooled AUC is a descriptive summary and does not establish broadly generalizable performance across data sources, ADR definitions, feature-engineering strategies, or validation settings. The high heterogeneity indicates substantial variation in underlying performance across contexts. Standardized benchmarks, harmonized outcome taxonomies, mandatory external validation, and regulator-aligned prospective evaluation are prerequisites for clinical deployment.

**Systematic Review Registration:**

[https://www.crd.york.ac.uk/PROSPERO/view/CRD420250653686], identifier [CRD420250653686].

## Introduction

Adverse drug reactions (ADRs) are a principal driver of preventable hospital admissions, iatrogenic morbidity, and in-hospital mortality, generating substantial direct cost, indirect cost, and disability-adjusted life-year burden across high- and middle-income health systems. Contemporary estimates of ADR-related hospital admissions range from approximately 5% in earlier systematic reviews (Kongkaew et al., 2008) to as high as 16.5% in recent prospective UK cohorts (Osanlou et al., 2022), with substantially higher burdens in polypharmacy, elderly, and oncology populations, and a disproportionate impact on patients on multi-drug oncological regimens or long-term immunosuppressive therapy. Despite decades of investment, the dominant pharmacovigilance paradigm remains reactive and based on spontaneous reporting, suffering from systematic under-reporting, latency between drug exposure and signal detection, selection bias, and limited sensitivity for rare or late-onset events (Sharrar and Dieck, 2013). Regulatory initiatives such as the United States Food and Drug Administration (FDA) Sentinel Real-World Evidence Data Enterprise (Desai et al., 2024) have begun to address these shortcomings by enabling active, large-scale real-world surveillance, but they remain fundamentally retrospective and dependent on adjudicated safety signals reaching the regulator.

Machine learning (ML) has emerged as a potentially transformative, data-driven alternative, possibly capable of predicting ADR. By integrating heterogeneous inputs, including chemical structures, pharmacological properties, electronic health record (EHR) phenotypes, and biological pathway annotations, ML algorithms can detect latent signals of ADR risk that elude classical signal-detection methods and can in principle issue early-warning predictions before a clinically actionable signal accumulates in passive surveillance (Rajpurkar et al., 2022; Lee and Chen, 2021). A substantial methodological literature has accumulated, including knowledge-graph embeddings, deep neural networks, and mechanistic feature integration with chemical-protein interaction descriptors (Zhang et al., 2021; Hauben et al., 2024). Yet three critical uncertainties persist that have so far prevented a coherent quantitative summary of the field.

First, there is no consensus on which model families offer the best discrimination versus interpretability trade-off in clinically realistic settings. Prior reviews have been either descriptive (Kim et al., 2022), restricted to specific methodological questions (Ribeiro-Vaz et al., 2016), or focused on model development without clinical-applicability criteria (Dsouza et al., 2025). Second, the assumption that incorporating biological mechanistic features such as protein targets and pathway annotations necessarily improves prediction has not been systematically tested (Hu et al., 2024). Third, prior pooled analyses have treated multiple models from the same study as independent observations, inflating apparent precision and potentially biasing subgroup comparisons (Yasrebi-de Kom et al., 2023; Christodoulou et al., 2019). A dependence-respecting synthesis is needed to translate the rapidly accumulating ML-ADR literature into evidence that is directly comparable across algorithm classes and feature sets, and that can inform regulatory and clinical decision-support design under the explainability and auditability constraints articulated by recent pharmacovigilance regulators (Pinheiro and Kurz, 2022).

We therefore aimed to provide the first PRISMA 2020 compliant, PROBAST-screened, random-effects and three-level multilevel meta-analytic synthesis of ML-based broad-spectrum ADR prediction under strict clinical-applicability criteria, and to formally test moderator effects of algorithm class, protein-target feature inclusion, and outcome breadth. The contribution of this review is a dependence-respecting quantitative synthesis that separates model-level from study-level variation and tests whether algorithm class, protein-target features, and outcome breadth explain performance differences. Recent drug-drug interaction side-effect prediction work has compared homogeneous and graph-based models, illustrating a related but distinct task outside our broad-spectrum ADR eligibility framework (Koç et al., 2025).

## Methods

### Protocol and registration

This systematic review and meta-analysis was conducted and reported in accordance with the PRISMA 2020 statement (Page et al., 2021) and its extension for searching, PRISMA-S (Rethlefsen et al., 2021). The protocol was prospectively registered in the International Prospective Register of Systematic Reviews (PROSPERO, CRD420250653686) prior to data extraction. The review question was framed using the Population, Intervention, Comparator, Outcome (PICO) framework (Schardt et al., 2007). No substantive protocol amendments were made after registration. Minor clarifications to the statistical analysis plan, namely, addition of the three-level multilevel meta-regression, were made prior to data lock and are documented in Supplementary Material S10. The end-to-end study pipeline is depicted in Figure 1. Reporting additionally followed PRISMA-DTA principles where applicable to prediction-model evidence and was benchmarked against the TRIPOD + AI checklist (Collins et al., 2024), while recognizing that TRIPOD + AI targets primary prediction-model studies rather than systematic reviews thereof.

**FIGURE 1:**
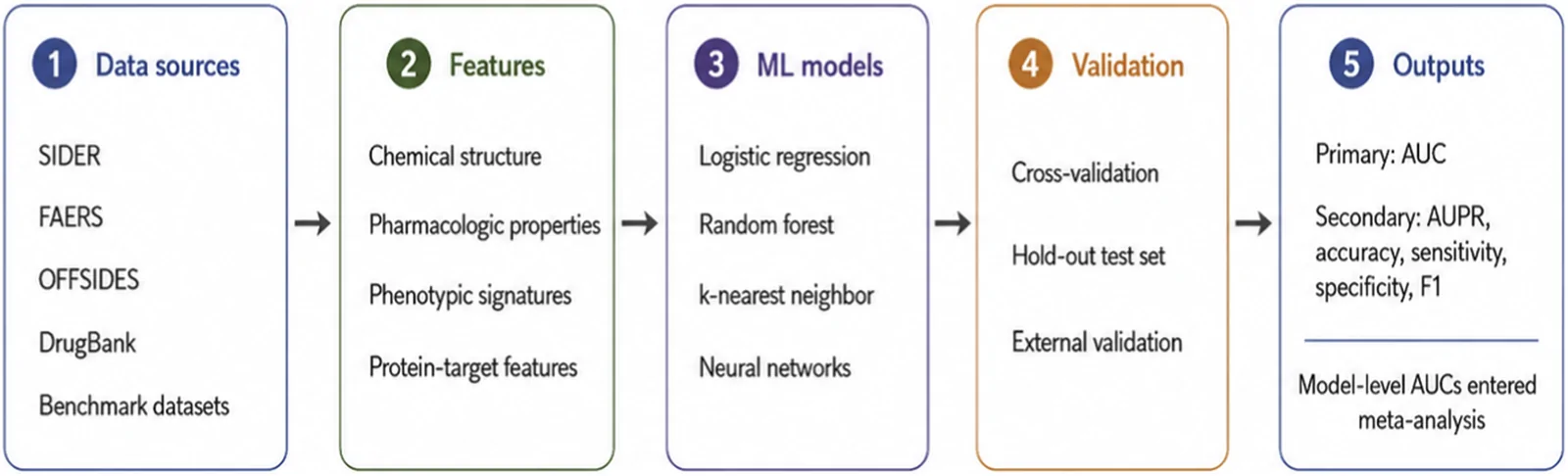
End-to-end machine-learning pipeline for adverse drug reaction prediction. The figure illustrates the analytical workflow used in this systematic review and three-level multilevel meta-analysis. Clinically validated data sources, including SIDER, FAERS, OFFSIDES, DrugBank, and benchmark datasets, were used to derive structured feature representations comprising chemical-structure descriptors, pharmacologic properties, phenotypic signatures, and protein-target information. These features were entered into supervised machine-learning models, including logistic regression, random forests, k-nearest-neighbor models, and neural-network-based approaches. Model performance was assessed using internal cross-validation, hold-out testing, or external validation where available. The primary performance metric was discrimination measured by the area under the receiver operating characteristic curve (AUC); secondary metrics included AUPR, accuracy, sensitivity, specificity, and F1 score. Model-level AUCs were subsequently entered into the quantitative synthesis. ADR denotes adverse drug reaction; AUC, area under the receiver operating characteristic curve; AUPR, area under the precision-recall curve.

### Eligibility criteria

Studies were eligible if they (i) were peer-reviewed original research articles published in English, (ii) developed or evaluated at least one supervised ML model for ADR prediction, (iii) used clinically validated data sources, including spontaneous-reporting databases such as the FDA Adverse Event Reporting System (FAERS) or the Side Effect Resource (SIDER), electronic health records, or curated chemical or biological databases of marketed drugs, (iv) reported sufficient performance metrics to permit quantitative extraction of AUC or equivalent discrimination measures, and (v) adhered to reproducible reporting standards (Collins et al., 2015). We excluded preclinical and animal studies, studies restricted to pharmacogenomic or genetic predictors, drug-drug interaction studies, studies restricted to a single drug or single therapeutic class, studies evaluating drugs not yet approved for clinical use, studies using unvalidated data sources such as social media, conference abstracts without peer-reviewed full texts, and narrative reviews, editorials, or letters. Studies predicting a single ADR across multiple drugs were retained and analyzed as the single-ADR subgroup, distinct from single-drug studies which were excluded. For this review, clinically applicable broad-spectrum ADR prediction meant prediction of one ADR across multiple marketed drugs or prediction of multiple ADRs across one or more marketed drugs using clinically validated data sources. Studies restricted to a single drug or therapeutic class were excluded. The complete PICO-D operationalization is provided in Supplementary Material S11.

### Information sources and search strategy

We searched PubMed/MEDLINE and Web of Science Core Collection from database inception to 30 January 2025. The search combined controlled vocabulary and free-text terms for two concept blocks. The first block covered machine learning, including ‘machine learning’, ‘deep learning’, ‘neural network*', and ‘*in silico*’. The second block covered adverse drug reactions, including ‘adverse drug reaction*', ‘side effect*', ‘drug toxicity’, and ‘adverse drug event*'. Complete Boolean strings are provided in Supplementary Material S3. Embase and ACM Digital Library were not searched. A targeted forward- and backward-citation screen of the 11 included studies and five prior reviews was performed on 5 March 2025, and a supplementary top-up search of Scopus and IEEE Xplore was executed on 2 February 2026 (Supplementary Material S3, Section 3). Neither identified additional eligible records. To reduce database-specific retrieval bias, the primary search was supplemented by forward- and backward-citation screening and later Scopus and IEEE Xplore searches. Embase and ACM Digital Library were not searched and remain a source of potential retrieval bias.

### Study selection and data extraction

Records were imported into Zotero version 7.0 and deduplicated. Two reviewers (G.C., J.G.) independently screened titles and abstracts. Full texts were assessed in duplicate (Cohen kappa 0.84). Disagreements were adjudicated by a senior reviewer (S.K.). Two reviewers (G.C., J.G.) independently extracted data into a pre-piloted form covering bibliographic details, data sources and sample sizes, feature-set composition (chemical structure descriptors, pharmacological properties, phenotypic signatures, protein-target interactions, EHR-derived features), ML algorithm and tuning strategy, prediction target (single-vs. multi-ADR), validation strategy, and performance metrics. The primary metric was the AUC of the receiver operating characteristic. Secondary metrics were the area under the precision-recall curve (AUPR), accuracy, sensitivity, specificity, F1, and calibration. Discrepancies were reconciled by consensus. The full PRISMA 2020 flow diagram is shown in Figure 2.

**FIGURE 2:**
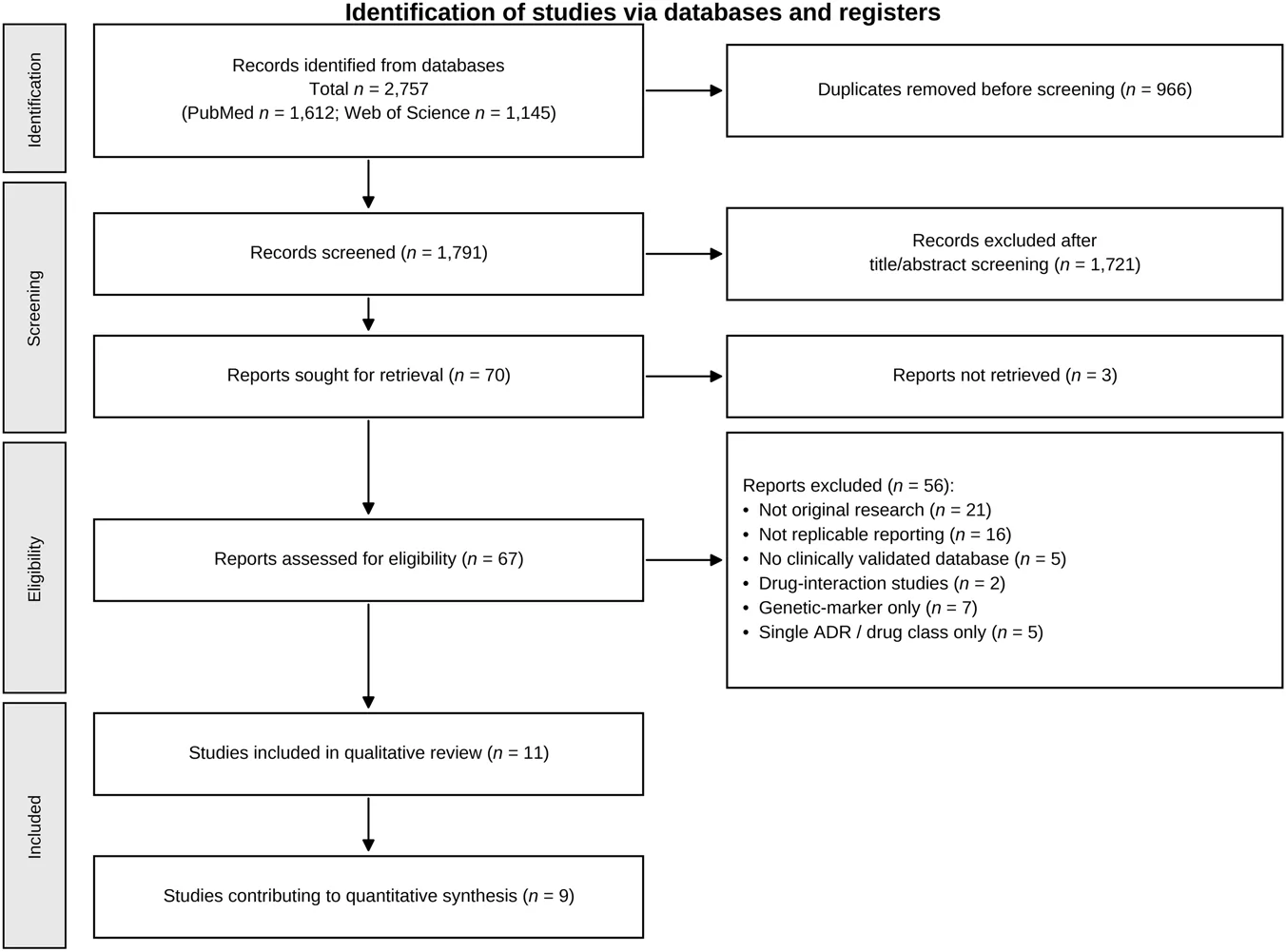
PRISMA 2020 study selection flow diagram.PRISMA 2020 flow diagram of study selection. Identification phase. 2,757 records were retrieved (1,612 PubMed, 1,145 Web of Science). Screening phase. 966 duplicates were removed, leaving 1,791 unique records. Of these, 1,721 were excluded after title and abstract screening. Eligibility phase. 70 full texts were assessed. Three could not be retrieved and 56 were excluded with reasons (21 non-original research, 16 not meeting reproducible reporting standards, 5 unvalidated data sources, 14 not reflecting real-world clinical applicability). Inclusion phase. 11 PROBAST-screened studies were retained for the systematic review, of which 9 contributed 30 model-level AUCs to the quantitative synthesis. Dubey and Pandit (2022) and Cao et al. (2024) reported only accuracy, precision, recall, and F1 and are retained in the descriptive synthesis only. AUC, area under the receiver operating characteristic curve. PRISMA, Preferred Reporting Items for Systematic Reviews and Meta-Analyses. PROBAST, Prediction model Risk Of Bias ASsessment Tool.

### Risk of bias assessment

Risk of bias was assessed independently in duplicate using the Prediction model Risk Of Bias ASsessment Tool, abbreviated PROBAST (Moons et al., 2019; Wolff et al., 2019), which evaluates four domains, namely, participants, predictors, outcome, and analysis. We pre-specified a primary synthesis restricted to studies at low or moderate risk in all four domains and additionally reported a sensitivity analysis including all studies regardless of risk of bias (Supplementary Material S6). Applicability concerns were assessed separately and did not lead to exclusions.

### Data synthesis and statistical analysis

The primary outcome was model discrimination quantified by the AUC. AUCs were logit-transformed to stabilize variance. The variance of each AUC was approximated using the Hanley-McNeil Formula (21), with test-set sample sizes extracted from each primary study where reported and, where not reported, imputed as the median of the reported sample sizes (Supplementary Material S9). As a pre-specified sensitivity analysis for comparability with the prior literature, we fitted a study-level DerSimonian-Laird random-effects meta-analysis (DerSimonian and Laird, Control Clin Trials, 1986, DOI: 10.1016/0197-2456(86)90046-2), in which each study contributed a single pooled logit-AUC calculated as the median of its reported models. Between-study heterogeneity was quantified by tau-squared and I-squared (Higgins et al., 2003). The pre-specified primary analysis was a three-level multilevel meta-analytic model, with models nested within studies, fitted by REML (Van den Noortgate et al., 2013; Assink and Wibbelink, 2016), which jointly estimates within-study and between-study variance components and appropriately propagates uncertainty from the model-level dependence structure, permitting inclusion of all 30 model-level AUCs without inflating precision. Where the two estimates disagreed, inference was anchored on the three-level multilevel model, which respects the data dependence structure. Convergence of the REML optimiser was verified. One non-positive-definite Hessian warning arose in the protein-target sub-model after exclusion of Dey et al. and is reported transparently in Supplementary Materials S4, S10. Two of the nine quantitatively contributing studies required test-set-size imputation, namely, Bongini et al., 2023 and Mantripragada et al., 2021. One endpoint from Cao et al., 2024 also lacked usable sample-size information, but Cao et al. did not contribute an AUC to the pooled analyses. The three-level model accounts for dependence among model-level estimates. It does not compensate for the limited number of independent studies available for estimating between-study variance or moderator effects.

Pre-specified sensitivity analyses included leave-one-study-out re-estimation (Figure 6C), restriction to studies at low risk of bias in all PROBAST domains, exclusion of the dominant single study (Dey et al.) for the protein-target subgroup analysis, and halved or doubled imputed sample-size analyses. Pre-specified moderator analyses were conducted via mixed-effects meta-regression with the following moderators, namely, (a) algorithm class (deep neural networks vs. traditional ML), (b) protein-target feature inclusion (binary), and (c) single-ADR vs. multi-ADR outcome. For comparability with the prior literature, unadjusted non-parametric comparisons (Kruskal–Wallis with Dunn-Bonferroni *post hoc*, Mann-Whitney U) are reported alongside the correctly specified mixed-effects results in Table 2 and Figure 4. Small-study effects were assessed via funnel plots (Figure 6B) and Egger regression. Certainty of evidence was graded using GRADE adapted for prognostic-model reviews (Schunemann et al., 2013; Foroutan et al., 2020). All analyses were conducted in Python 3.13.0 with SciPy 1.14.0, statsmodels 0.14.2, and scikit-posthocs 0.9.1, and cross-validated in R 4.4.1 using the metafor package version 4.6-0 (Viechtbauer, 2010) via rma and rma.mv. Analysis code is available from the corresponding author on reasonable request. Algorithm-class findings were reported at both the prespecified grouped level and the individual-algorithm level. Further granular moderator modeling was not undertaken because only nine studies contributed to the quantitative synthesis and additional parameters would be statistically unstable.

## Results

### Study selection

The database searches yielded 2,757 records, comprising 1,612 from PubMed and 1,145 from Web of Science. After removal of 966 duplicates, 1,791 unique records remained. After title and abstract screening, 70 full texts were assessed for eligibility. Three could not be retrieved, and 56 were excluded with reasons (21 non-original research, 16 not meeting reproducible reporting standards, 5 unvalidated data sources, 14 not reflecting real-world clinical applicability). The final analytical sample comprised 11 PROBAST-screened eligible studies. Nine studies contributed 30 model-level AUCs to the quantitative synthesis. Dubey and Pandit (Dubey and Pandit, 2022) and Cao et al. (Cao et al., 2024) reported only accuracy, precision, recall, and F1 without discrimination metrics and therefore contribute to the descriptive synthesis (Table 1 and Supplementary Material S1) but not to the pooled quantitative analyses (Figure 2).

**TABLE 1:** Characteristics of included studies.Per-study extraction of design and ML-modeling characteristics for the 11 PROBAST-eligible studies. Columns capture country, algorithm class, feature set, prediction outcome (single-ADR or multi-ADR), number of model-level AUCs supplied to the quantitative synthesis, and the reported AUC range or point estimate. Studies marked with an asterisk, namely, Dubey and Pandit (Dubey and Pandit, 2022) and Cao et al. (Cao et al., 2024), were retained as PROBAST-eligible for descriptive completeness but contribute zero quantitative model-level AUCs because the studies report only accuracy, precision, recall, and F1 and provide no extractable AUC or AUPR. The pooled quantitative meta-analysis therefore synthesizes 30 model-level AUCs nested within 9 of the 11 PROBAST-eligible studies. Full per-study extraction is provided in Supplementary Material S1. ADR, adverse drug reaction. AUC, area under the receiver operating characteristic curve. AUPR, area under the precision-recall curve.

Study	Country	Algorithm class	Features	Outcome	Models (n; quant.)	Reported AUC
Jamal et al., 2017	India	SMO/SVM	Chemical substructures + biological properties	Single-ADR (neurological)	7 (incl.)	0.49–0.74
Munoz et al., 2019	Ireland	MLP/KNN/decision tree/random forest	Protein targets + chemical + EHR	Multi-ADR	4 (incl.)	0.63–0.88
Dey et al., 2018	USA	Neural fingerprints (DNN)	Chemical-structure fingerprints	Multi-ADR (1,766 SIDER terms)	10 (incl.)	0.90–0.96
Wang et al., 2019	Taiwan	DNN (2 hidden layers)	Molecular substructures + biological properties	Multi-ADR	1 (incl.)	0.844
Amano et al., 2020	Japan	L1 logistic regression	Chemical + chemical-protein interactions	Multi-ADR (CV/GI/CNS)	4 (incl.)	0.64–0.70
Galeano et al., 2020	UK/Brazil/USA	Matrix decomposition + K-means	Chemical + target proteins	Multi-ADR (SIDER 4.1)	1 (incl.)	0.932
Mantripragada et al., 2021	India	CNN (DCNN)	Chemical-structure fingerprints + protein-target descriptors	Single-ADR	1 (incl.)	0.896
Dubey and Pandit, 2022 *	India	DNN (1–3 hidden layers)	FAERS + SIDER + PubChem + DrugBank	Single-ADR	0 (descriptive only*)	Not extractable
Yu et al., 2022	China	Random forest (ADENet)	Chemical + target proteins	Multi-ADR	1 (incl.)	0.917
Bongini et al., 2023	Italy	Graph neural network	SMILES/molecular graph + protein-target node attributes (DruGNN)	Single-ADR	1 (incl.)	0.867
Cao et al., 2024 *	Taiwan	Multilayer perceptron	Target proteins + IC50/pIC50	Single-ADR (4 events)	0 (descriptive only*)	Not extractable

### Characteristics of included studies

Included studies were published between 2017 and 2024 and spanned a diverse range of data sources (SIDER, FAERS, OFFSIDES, DrugBank, Liu et al. benchmark, Bio2RDF, Aeolus) and feature modalities, including chemical-structure fingerprints, pharmacological properties, phenotypic signatures, protein-target interactions, and combinations thereof. Study-level characteristics are summarized in Table 1, with the full extraction in Supplementary Material S1. Neural-network-based models (multilayer perceptron [MLP], convolutional neural network [CNN], graph neural network [GNN], neural-fingerprint frameworks) were the most frequently employed algorithmic class. Logistic regression and random forests each appeared in two studies.

### Risk of bias

Of 14 full-text-eligible studies initially considered, three were excluded for high risk of bias in the PROBAST analysis domain (inadequate missing-data handling in two, no overfitting-prevention strategy in one), leaving 11 PROBAST-screened studies that entered the review; these three are counted within the 56 excluded reports shown in Figure 2. Of these, 9 contributed to the quantitative synthesis, while Dubey and Pandit (2022) as well as Cao et al. (2024) remained descriptive only because no discrimination metric (AUC or AUPR) was extractable. Across the 11 PROBAST-screened studies, the analysis domain was rated moderate in 6 of 11 (insufficient calibration assessment in all six and limited sample-size justification in three), high in 1 of 11, namely, Dubey and Pandit (2022), and low in 4 of 11. The overall PROBAST rating was accordingly moderate in six studies, low in four studies, and high in the single study Dubey and Pandit (2022), which was excluded from the pooled quantitative synthesis. No study was rated high risk in the participants, predictors, or outcome domains, and applicability concerns were low overall (domain-level breakdown in Supplementary Material S5).

### Primary outcome: pooled discrimination (AUC)

The DerSimonian-Laird random-effects meta-analysis of 9 study-level AUCs yielded a pooled AUC of 0.832 (95% CI 0.788-0.869, tau-squared 0.21, I-squared 90.7%, Cochran Q 107.4, p < 0.0001), pre-specified as a sensitivity analysis. The pre-specified primary three-level multilevel meta-analysis (30 model-level observations nested in 9 studies) yielded a pooled AUC of 0.841 (95% CI 0.789-0.883), with tau-squared between of 0.20 and tau-squared within of 0.23 (level-3 I-squared approximately 46%). The larger total variance recovered by the multilevel model (tau-squared total approximately 0.43), compared with the study-level DerSimonian-Laird estimate (tau-squared 0.21), is expected, because the three-level model additionally captures within-study heterogeneity across the multiple ML models reported by the same study. Leave-one-study-out estimates ranged from 0.818 to 0.842 (Figure 6C). The funnel plot was approximately symmetric, and Egger regression did not indicate significant small-study effects (intercept 0.42, p = 0.31, Figure 6B). The I-squared of 90.7% indicates substantial variation in underlying model performance across contexts. The pooled estimate therefore has limited value as a single expectation for a new clinical setting. Test-set size was imputed for Bongini et al., 2023 and Mantripragada et al., 2021. Across the prespecified imputation scenarios, the primary pooled AUC ranged from 0.836 to 0.846 and the DerSimonian-Laird estimate ranged from 0.828 to 0.837 (Supplementary Material S9).

### Algorithm class: deep neural networks versus traditional ML

Table 2 presents the mean AUC ranked by algorithm class, with the full distribution in Figure 3 and per-algorithm granular ranking in Table 3. The highest mean AUC (0.932) was achieved by a hybrid matrix-decomposition and k-means clustering approach. The lowest mean AUC (0.627) was achieved by a sequential minimal optimization (SMO) support-vector implementation. Unadjusted non-parametric comparisons indicated overall differences (Kruskal–Wallis H 21.7, p = 0.0011), with *post hoc* Dunn-Bonferroni tests identifying neural networks as significantly outperforming SMO (adjusted p = 0.001, Figure 4). However, in the independence-respecting mixed-effects meta-regression the effect of algorithm class on logit-AUC was attenuated and no longer statistically significant, although the point estimate continued to favor neural networks (adjusted beta 0.31, 95% CI -0.08 to 0.70, p = 0.12). The absence of statistically significant differences in the mixed-effects meta-regression does not establish comparable performance across algorithm classes. With only nine contributing studies, the moderator analysis had limited statistical power and is exploratory.

**TABLE 2:** Mean AUC ranked by algorithm class. Algorithm-class ranking by mean AUC across all 30 model-level AUCs from the 9 quantitatively contributing studies. Columns report number of contributing models per class, mean AUC, standard deviation, and the unadjusted Dunn-Bonferroni *post hoc* adjusted p-value versus sequential minimal optimization as reference. The accompanying inference reported in the main text comes from the dependence-respecting three-level mixed-effects meta-regression, in which algorithm class was not a statistically significant moderator (adjusted beta 0.31, 95% CI -0.08 to 0.70, p = 0.12). AUC, area under the receiver operating characteristic curve. SD, standard deviation. SMO, sequential minimal optimization.

Algorithm class	k (models)	Mean AUC	SD	Adjusted p vs. SMO
Neural networks (DNN, CNN, GNN, MLP, neural fingerprints)	14	0.898	0.052	0.001
Matrix decomposition/clustering	1	0.932	–	0.02
Random forest	2	0.863	0.054	0.04
k-nearest neighbor	1	0.884	–	0.08
Logistic regression (L1-regularised)	4	0.670	0.025	0.18
Decision tree	1	0.630	–	0.25
Sequential minimal optimisation (SMO)	7	0.627	0.077	ref

**FIGURE 3:**
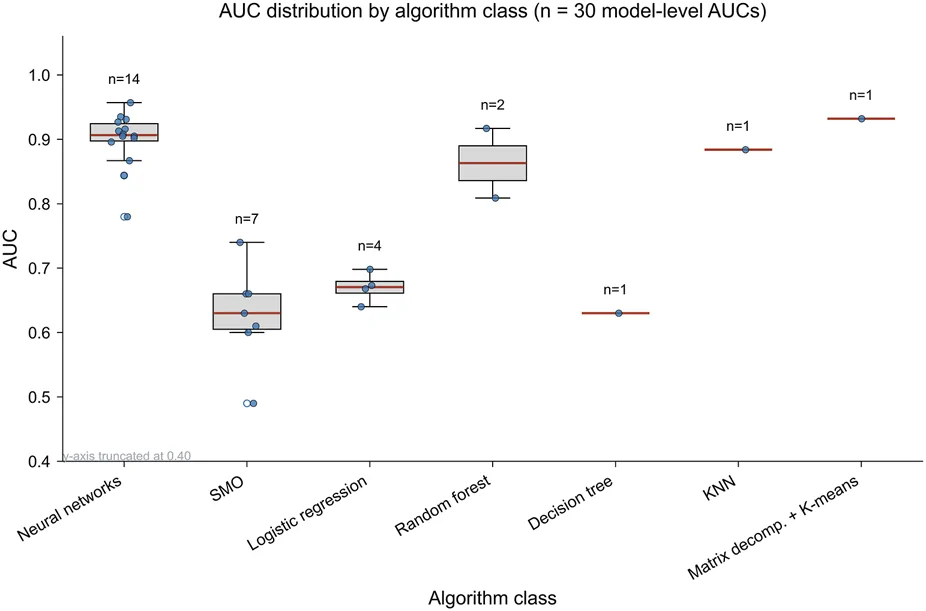
AUC distribution by algorithm class across all included models. Distribution of AUC scores by algorithm class across all 30 quantitatively contributing model-level AUCs. Box plots show the median as a horizontal line, the box as the interquartile range, whiskers as 1.5 times the interquartile range, and individual model-level AUCs overlaid as dots. Algorithm classes shown comprise neural networks, matrix decomposition with k-means clustering, random forest, k-nearest neighbor, logistic regression, decision tree, and sequential minimal optimization. Inference reported in the main text comes from the dependence-respecting three-level mixed-effects meta-regression, in which the algorithm-class effect was not statistically significant (adjusted beta 0.31, 95% CI -0.08 to 0.70, p = 0.12). AUC, area under the receiver operating characteristic curve. CNN, convolutional neural network. GNN, graph neural network. KNN, k-nearest neighbor. MLP, multilayer perceptron. SMO, sequential minimal optimization.

**TABLE 3:** Mean AUC by individual machine-learning algorithm.Granular per-algorithm ranking across all 30 quantitatively contributing model-level AUCs from 9 studies. Each row corresponds to one specific algorithm implementation (for example, matrix decomposition with k-means clustering, neural fingerprints, convolutional neural network, graph neural network, deep neural network with two hidden layers, k-nearest neighbor, random forest, multilayer perceptron, L1-regularised logistic regression, decision tree, and sequential minimal optimization), with the number of contributing models per implementation and the mean AUC. Dubey and Pandit (Dubey and Pandit, 2022) and Cao et al. (Cao et al., 2024) are not represented because no AUC is reported. AUC, area under the receiver operating characteristic curve.

Machine-learning algorithm	k (models)	Mean AUC
Matrix decomposition + K-means clustering (Galeano et al., 2020)	1	0.932
Neural fingerprints (Dey et al., 2018)	10	0.920
Convolutional neural network (Mantripragada et al., 2021)	1	0.896
k-nearest neighbor (Munoz et al., 2019)	1	0.884
Graph neural network (Bongini 2023)	1	0.867
Random forest (Munoz et al., 2019; Yu et al., 2022)	2	0.863
Deep neural network, 2 hidden layers (Wang et al., 2019)	1	0.844
Multilayer perceptron (Munoz et al., 2019)	1	0.780
L1-regularised logistic regression (Amano et al., 2020)	4	0.670
Decision tree (Munoz et al., 2019)	1	0.630
Sequential minimal optimisation/SMO-SVM (Jamal et al., 2017)	7	0.627

**FIGURE 4:**
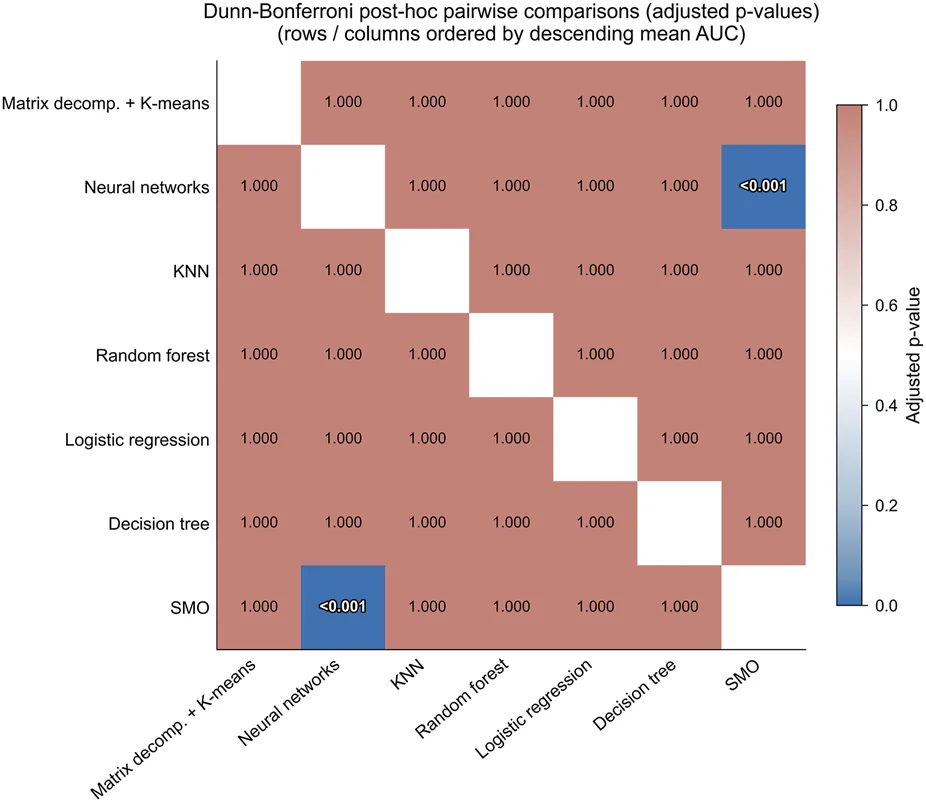
Dunn-Bonferroni *post hoc* pairwise comparison heatmap across algorithm classes. Post-hoc pairwise Dunn-Bonferroni comparison heatmap across algorithm classes. Each cell encodes the adjusted p-value for the pairwise contrast between two algorithm classes. Red shading (adjusted p approximately 1.000) indicates no significant pairwise difference, blue shading (adjusted p < 0.05) indicates a significant pairwise difference. The neural-network versus sequential minimal optimization contrast was the only statistically significant pairwise comparison at the unadjusted model level. Tests were Dunn pairwise *post hoc* with Bonferroni correction following an overall Kruskal–Wallis test (H 21.7, p = 0.0011). The figure should be interpreted alongside Figure 3 and Table 2, since the dependence-respecting three-level mixed-effects meta-regression in the main text indicated that algorithm class is not a statistically significant moderator (adjusted beta 0.31, 95% CI -0.08 to 0.70, p = 0.12). AUC, area under the receiver operating characteristic curve.

### Protein-target feature contribution

After re-extraction, eight of the 9 quantitatively contributing studies incorporated protein-target interaction features and contributed 20 of the 30 models, while the remaining single study, Dey et al. (Dey et al., 2018), contributed all 10 non-protein-target models. Dubey and Pandit (Dubey and Pandit, 2022) and Cao et al. (Cao et al., 2024) are not included in this analysis because no AUC was reported. The Feature column in Table 1 shows the primary input encoding per study. Bongini et al. (Bongini et al., 2023) use a SMILES or molecular graph and Mantripragada et al. (Mantripragada et al., 2021) use chemical-structure fingerprints, but both additionally integrate protein-target information, namely, target-protein node attributes in the DruGNN architecture and protein-target interaction descriptors alongside the chemical-structure input, as specified in Supplementary Material S2. Both studies are therefore classified as protein-target-positive in Figure 5 and the moderator analysis. Unadjusted model-level analysis suggested that models excluding protein-target features achieved higher mean AUC (0.919 vs. 0.731, Mann-Whitney U p = 0.0002, Figure 5). This signal was, however, fully attributable to a single dominant study. At the study level the pooled AUC difference was not statistically significant (0.886 vs. 0.818, Wald z −1.36, p = 0.17), and in the three-level multilevel meta-regression excluding Dey et al. the effect vanished entirely (adjusted beta on logit-AUC -0.22, 95% CI -1.59 to 1.14, p = 0.75). The apparent association was dominated by study-level confounding and provides very low-certainty evidence about the independent contribution of protein-target features (sensitivity panel, Figure 6A).

**FIGURE 5:**
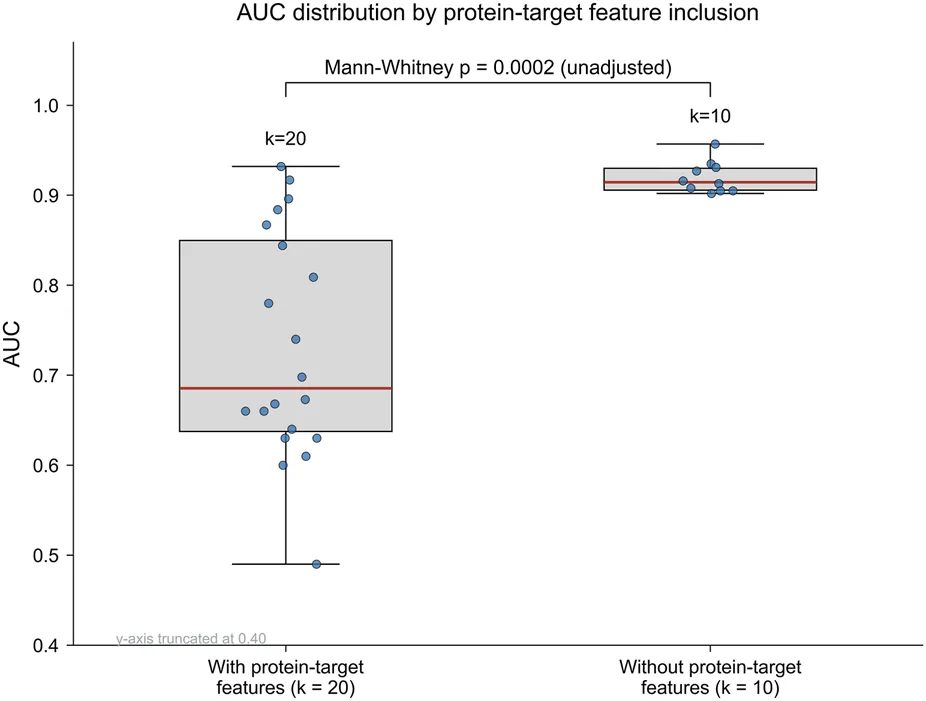
AUC distribution by protein-target feature inclusion. Distribution of AUC scores for models including (k = 20) versus excluding (k = 10) protein-target features. Box plots show the median as a horizontal line, the box as the interquartile range, and individual model-level AUCs overlaid as dots. All 10 non-protein-target models originate from a single dominant study (Dey et al., 2018), illustrating the confound that is addressed by the multilevel sensitivity analysis in Figure 6A. The unadjusted Mann-Whitney U test indicated higher AUCs in the protein-target-negative subset (mean 0.919 vs. 0.731, p = 0.0002), but this signal was fully attributable to single-study dominance. After exclusion of Dey et al., 2018 in the three-level mixed-effects meta-regression, the effect vanished entirely (adjusted beta on logit-AUC -0.22, 95% CI -1.59 to 1.14, p = 0.75). AUC, area under the receiver operating characteristic curve.

**FIGURE 6:**
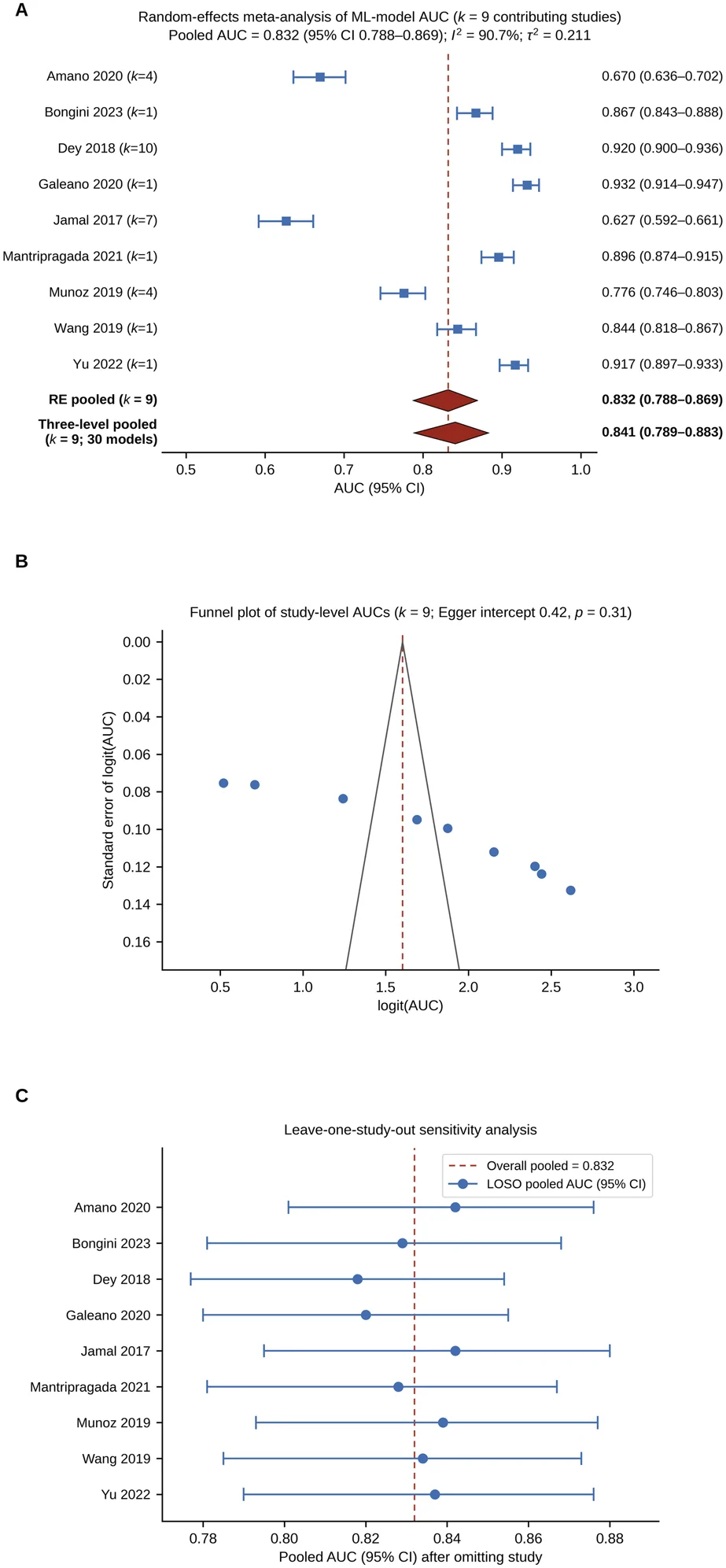
Random-effects and three-level multilevel meta-analysis of ML model discrimination for ADR prediction.Three-panel synthesis of the primary discrimination meta-analysis. **(A)** Forest plot showing study-level AUCs and 95% confidence intervals from the DerSimonian-Laird random-effects model (k = 9 quantitatively contributing studies, pooled AUC 0.832, 95% CI 0.788-0.869, I-squared 90.7%, tau-squared 0.21). Dubey and Pandit (Dubey and Pandit, 2022) and Cao et al. (Cao et al., 2024) are excluded because no AUC was reported. The pre-specified primary three-level multilevel pooled AUC of 0.841 (95% CI 0.789-0.883, 30 model-level AUCs nested in 9 quantitatively contributing studies) is shown as the bottom diamond. **(B)** Funnel plot of logit-transformed AUCs against standard error, showing approximate symmetry and no strong evidence of small-study effects (Egger regression intercept 0.42, p = 0.31, statistically underpowered at k = 9). **(C)** Leave-one-study-out sensitivity analysis demonstrating robustness of the pooled DerSimonian-Laird sensitivity estimate (range 0.818–0.842) when each of the 9 quantitatively contributing studies is omitted in turn. ADR, adverse drug reaction. AUC, area under the receiver operating characteristic curve. CI, confidence interval.

### Single-versus multi-ADR prediction

After reclassification of Dey et al., 2018 as a multi-ADR study because it simultaneously predicts across 1,766 SIDER side-effect terms, five studies predicted a single clinical ADR across multiple drugs and six predicted multiple ADRs (Table 4, study-level mapping in Table 5). Of these, three single-ADR studies and six multi-ADR studies contributed extractable AUCs to the quantitative synthesis. Single-ADR models (k = 9) achieved a mean AUC of 0.684 with median 0.660 and SD 0.130, and multi-ADR models (k = 21) achieved a mean AUC of 0.842 with median 0.905 and SD 0.112. The lower mean of the single-ADR group reflects the dominance of the seven low-performing SMO models of Jamal et al., with AUC range 0.49–0.74 (Jamal et al., 2017), and is only partially offset by the high-performing single model of Mantripragada et al. with AUC 0.896 (Mantripragada et al., 2021), rather than reflecting a data-extraction artifact (Figure 7). At the model level the unadjusted Mann-Whitney U test was significant (p = 0.002), driven by the strong clustering of the 10 high-AUC Dey et al. neural-fingerprint models within the multi-ADR group. Once this within-study dependence was respected in the three-level mixed-effects moderator analysis, no statistically significant difference between single- and multi-ADR prediction remained (adjusted beta on logit-AUC -0.21, 95% CI -1.09 to 0.68, adjusted p = 0.52). Multi-ADR studies used highly heterogeneous outcome definitions. Munoz et al. grouped ADRs by source database, namely, 5,549 SIDER, 1,385 Liu-set, and 5,580 Bio2RDF (Munoz et al., 2019). Yu et al. used 26 Medical Subject Headings derived categories, abbreviated MeSH (Yu et al., 2022). Wang et al. simultaneously predicted 1,325 ADRs (Wang et al., 2019). Galeano et al. targeted the full SIDER 4.1 taxonomy with 5,880 ADRs (Galeano et al., 2020). Amano et al. used three organ-system categories (Amano et al., 2020). This heterogeneity precludes meaningful quantitative synthesis of multi-ADR performance.

**TABLE 4:** Single-ADR versus multi-ADR model performance after reclassification of Dey et al. 2018 as multi-ADR.Aggregated model-level performance by outcome breadth after reclassification of Dey et al., 2018 as multi-ADR (because the model simultaneously predicts across 1,766 SIDER side-effect terms). Columns report the number of contributing studies, the number of model-level AUCs, mean AUC, median AUC, and standard deviation. Dubey and Pandit (Dubey and Pandit, 2022) is tabulated within the single-ADR study count for completeness but contributes zero quantitative model-level AUCs. Unadjusted Mann-Whitney U at the model level was significant (p approximately 0.002), driven by within-study clustering of the 10 Dey et al. neural-fingerprint models. Respecting model-level dependence in the three-level mixed-effects moderator analysis, no statistically significant difference remained (adjusted beta on logit-AUC -0.21, 95% CI -1.09 to 0.68, adjusted p = 0.52). ADR, adverse drug reaction. AUC, area under the receiver operating characteristic curve. SD, standard deviation.

Outcome type	k (studies)	k (models)	Mean AUC	Median AUC	SD
Single-ADR	5 (Jamal, Mantripragada, Bongini, + Dubey & Cao descriptive*)	9	0.684	0.660	0.130
Multi-ADR	6 (Munoz, Dey, Wang, Amano, Galeano, Yu)	21	0.842	0.905	0.112

**TABLE 5:** Study-level classification of single- versus multi-ADR prediction models. Per-study classification as single-ADR or multi-ADR after the extraction-clarification reclassification of Dey et al. (2018) as multi-ADR (simultaneous prediction across 1,766 SIDER side-effect terms). The Quantitative contribution column indicates whether the study supplied extractable AUCs to the pooled meta-analytic models. Dubey and Pandit (2022) and Cao et al. (2024) are classified as single-ADR for descriptive completeness but contribute 0 model-level AUCs because no AUC is reported. ADR, adverse drug reaction. AUC, area under the receiver operating characteristic curve.

Study	Prediction type	Rationale	Quant. contribution
Jamal et al., 2017	Single-ADR	Neurological ADR outcome	Yes
Munoz et al., 2019	Multi-ADR	12,514 side-effect terms (Liu/SIDER-4/Aeolus/Bio2RDF)	Yes
Dey et al., 2018	Multi-ADR	Simultaneous prediction across 1,766 SIDER terms	Yes
Wang et al., 2019	Multi-ADR	Broad ADR panel from MEDLINE	Yes
Amano et al., 2020	Multi-ADR	61 side-effect terms (CV/GI/CNS)	Yes
Galeano et al., 2020	Multi-ADR	Full SIDER 4.1 taxonomy (5,880 terms)	Yes
Mantripragada et al., 2021	Single-ADR	Single ADR outcome	Yes
Dubey and Pandit, 2022	Single-ADR	Single ADR presence/absence	No, descriptive only (no AUC reported)
Yu et al., 2022	Multi-ADR	ADENet across multiple ADRs	Yes
Bongini et al., 2023	Single-ADR	Per-drug single-label side-effect prediction	Yes
Cao et al., 2024	Single-ADR	Four discrete clinical adverse events	No, descriptive only (no AUC reported)

**FIGURE 7:**
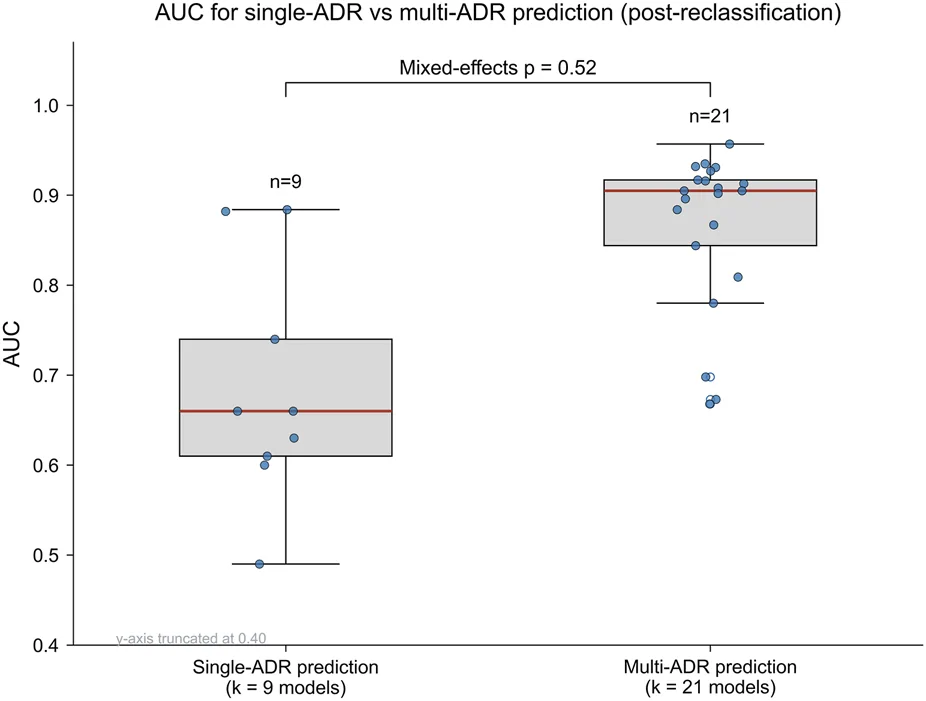
AUC distribution for single-ADR versus multi-ADR prediction models after reclassification of Dey et al. 2018 as multi-ADR. Distribution of AUC scores for single-ADR versus multi-ADR prediction models after reclassification of Dey et al., 2018 as multi-ADR (single-ADR k = 9, mean 0.684, median 0.660. Multi-ADR k = 21, mean 0.842, median 0.905). Box plots show the median, interquartile range, and individual model-level AUCs overlaid as dots. The lower mean of the single-ADR group is driven by the seven low-performing sequential minimal optimization models of Jamal et al. (2017) (AUC range 0.49–0.74) and is only partially offset by the high-performing single model of Mantripragada et al., 2021 (AUC 0.896), illustrating intra-group variability rather than a data-extraction artifact. The dependence-respecting three-level mixed-effects moderator analysis indicated no statistically significant difference between single- and multi-ADR outcome (adjusted beta on logit-AUC -0.21, 95% CI -1.09 to 0.68, adjusted p = 0.52). ADR, adverse drug reaction. AUC, area under the receiver operating characteristic curve.

### Certainty of evidence (GRADE)

Certainty of evidence was rated low for the overall pooled AUC estimate, downgraded for substantial inconsistency (I-squared above 90%) and risk of bias in the analysis domain. Certainty was rated low for the algorithm-class subgroup comparison, downgraded for heterogeneity and imprecision. Certainty was rated very low for the protein-target subgroup comparison, downgraded for indirectness, risk of bias, and single-study dominance. The full GRADE summary of findings is presented in Table 6.

**TABLE 6:** GRADE summary of findings. GRADE summary of findings for the four key outcomes of the synthesis, namely, overall pooled discrimination from the primary three-level multilevel REML model, overall pooled discrimination from the study-level DerSimonian-Laird sensitivity model, the protein-target feature effect after exclusion of Dey et al., and the single-ADR versus multi-ADR comparison. Pooled effect sizes, certainty ratings, downgrading reasons, and importance designations are reported per row. All quantitative rows exclude Dubey and Pandit (Dubey and Pandit, 2022) because no AUC was extractable. Certainty was downgraded once for inconsistency and once for risk of bias. CI, confidence interval. DL, DerSimonian-Laird. GRADE, Grading of Recommendations Assessment, Development and Evaluation. REML, restricted maximum likelihood. RoB, risk of bias.

Outcome	Studies (models)	Pooled AUC (95% CI)	Certainty (GRADE)	Downgraded for	Importance
Overall discrimination, primary three-level multilevel REML	9 (30)	0.841 (0.789–0.883)	Low	Inconsistency (level-3 I^2^ ≈ 46%; I^2^ > 90% under DL sensitivity), risk of bias in PROBAST analysis domain (6/11 moderate, 1/11 high; high-RoB study excluded, see Table 1 footnote for full citation)	Critical
Overall discrimination, study-level DerSimonian–Laird sensitivity	9 (9)	0.832 (0.788–0.869)	Low	Inconsistency, risk of bias	Critical
Protein-target feature effect (excl. Dey et al.)	8 (20)	Δ logit-AUC −0.22 (−1.59 to 1.14)	Very low	Risk of bias, inconsistency, indirectness, single-study dominance	Important
Single- vs. multi-ADR outcome	9 (30); 3 single vs. 6 multi quant	Not significant (adj. β −0.21; p = 0.52)	Low	Indirectness, imprecision	Important

## Discussion

This PRISMA 2020 compliant systematic review and three-level multilevel meta-analysis identified 11 PROBAST-eligible studies and synthesized 30 ML models from 9 of them quantitatively for ADR prediction under stringent clinical-applicability criteria. Four principal findings emerged. The pooled AUC was 0.832 with 95% CI 0.788-0.869 and a consistent three-level multilevel estimate of 0.841 with 95% CI 0.789-0.883, indicating moderate discrimination overall under GRADE low certainty. Unadjusted comparisons suggested that neural networks outperformed other algorithm classes, but this effect was attenuated and not statistically significant in the independence-respecting mixed-effects meta-regression. The widely cited advantage of protein-target features was not confirmed, because the apparent signal at p = 0.0002 was fully driven by one dominant study, Dey et al. (Dey et al., 2018), and vanished after its exclusion at adjusted p = 0.75. Leave-one-study-out estimates with pooled AUC range 0.82–0.84 showed numerical stability within the analyzed dataset. The small number of studies and marked heterogeneity limit precision and generalizability, while multi-ADR prediction was characterized by profound outcome-definition heterogeneity that precludes meaningful cross-study comparison.

The principal limitation is the small number of independent studies. Only nine studies contributed to the quantitative synthesis, heterogeneity was marked, and study-level variance and moderator estimates were consequently imprecise. Although the three-level model accounts for dependence among model-level estimates, 30 model-level observations do not compensate for only nine independent studies. Between-study variance estimates from REML and moderator estimates are therefore imprecise. Reporting of training-set scale, data quality, feature construction, and validation procedures was incomplete across studies. These omissions prevented reliable adjustment for several plausible sources of performance variation. Substantial heterogeneity in datasets, feature engineering, ADR definitions, and validation strategies complicates direct cross-study comparison (Christodoulou et al., 2019). Variances of AUC estimates were approximated via the Hanley-McNeil formula using reported or imputed test-set sizes; halved and doubled imputed-sample-size sensitivity analyses did not materially alter the pooled estimate (Supplementary Material S9). Reliance on AUC as the primary outcome provides an incomplete view of clinical utility, because calibration, sensitivity, specificity, and positive predictive value were inconsistently reported (Alba et al., 2017). Embase and ACM Digital Library were not searched, although a Scopus and IEEE Xplore top-up on 2 February 2026 and a forward- and backward-citation screen identified no additional eligible records. Publication bias cannot be entirely excluded despite a symmetric funnel plot, because Egger regression is statistically underpowered at k = 9. The application of GRADE to prognostic-model reviews remains methodologically evolving (Foroutan et al., 2020), and none of the included studies adhered to the TRIPOD + AI reporting standard (Collins et al., 2024).

To our knowledge, this is the first systematic review of ML-based broad-spectrum ADR prediction that combines pre-registered PROBAST risk-of-bias exclusion under the unified Wolff and Moons criteria (Moons et al., 2019; Wolff et al., 2019), a formal random-effects meta-analysis, a dependence-respecting three-level multilevel meta-regression fitted by REML through both statsmodels and the metafor R package (Viechtbauer, 2010), pre-specified sensitivity analyses, and GRADE certainty rating. Search reporting was conducted in line with PRISMA-S (Rethlefsen et al., 2021). The agreement between the study-level and multilevel point estimates and the leave-one-study-out range indicates numerical stability within the analyzed dataset. It does not overcome the limited number of studies or marked heterogeneity. The cleaned datasets are provided as Supplementary Materials S7 and S8, and the analysis code is available from the corresponding author on reasonable request.

Our pooled estimate is consistent with prior, less methodologically conservative syntheses. Hu et al. (Hu et al., 2024) reported pooled sensitivity and specificity of approximately 0.71 and 0.79 in EHR-based ML ADR prediction. Dsouza et al. observed pooled sensitivity and specificity of approximately 78% and 71% in hospitalized patients (Dsouza et al., 2025). Crucially, the present analysis demonstrates that the apparent superiority of deep-learning approaches over traditional ML is not statistically robust once study-level dependence is accounted for, aligning with accumulating evidence that well-specified logistic regression and gradient-boosted trees can match or exceed deep neural networks when feature sets are well curated (Christodoulou et al., 2019; Rajkomar et al., 2019). The most consequential finding is the collapse of the protein-target feature effect under appropriate statistical handling. Recent knowledge-graph and mechanistic-integration approaches have implicitly assumed that richer biological annotation must improve prediction (Zhang et al., 2021; Hauben et al., 2024), but our multilevel analysis raises the alternative hypothesis that protein-target data, when incompletely curated or imperfectly mapped to clinical ADR phenotypes, introduces noise and promotes overfitting without proportional gain (Liu et al., 2012). The available studies did not report training-set scale and data quality consistently enough to assess whether algorithm-class effects vary by data regime. The nonsignificant neural-network contrast cannot be generalized to larger or higher-quality training datasets.

Most urgently, the field requires standardized benchmarking frameworks with consensus datasets, harmonized ADR outcome taxonomies aligned with the Medical Dictionary for Regulatory Activities (MedDRA), and uniform performance reporting (Yasrebi-de Kom et al., 2023). External validation on independent, geographically and temporally diverse datasets must become a prerequisite for publication (Collins et al., 2015). Current evidence is insufficient to support preferential clinical integration of one algorithm class. Model selection should be guided by external validation, calibration, interpretability, and implementation context pending prospective evaluation (Pinheiro and Kurz, 2022). Real-world implementation should be aligned with regulatory infrastructure such as the FDA Sentinel Real-World Evidence Data Enterprise (Desai et al., 2024). The transition from retrospective, internally validated models to prospective, registry-based evaluation is the critical next step. The present estimates describe retrospective model discrimination and do not establish clinical utility or readiness for implementation. Translation requires externally validated and prospectively evaluated models with calibration and decision-analytic assessment.

In summary, this review provides a dependence-respecting quantitative synthesis of a limited and heterogeneous evidence base. The primary three-level pooled AUC was 0.841 (95% CI 0.789-0.883) under GRADE low certainty and should be interpreted as a descriptive summary. The absence of statistically significant algorithm-class differences does not establish comparable performance, and the protein-target finding carries very low certainty because it is dominated by one study. These estimates do not establish broadly generalizable performance or clinical utility. Progress toward clinical deployment requires standardized benchmarks, harmonized outcome taxonomies, complete reporting of training data and validation procedures, mandatory external validation, and regulator-aligned prospective evaluation.

## Data Availability

All data extracted from the 11 PROBAST-screened studies, including the descriptive-only entries for Dubey and Pandit (2022) and Cao et al. (2024) for which no discrimination metric is reported, the full database-specific search strings, the cleaned AUC datasets, and the PROBAST risk-of-bias assessments are provided as Supplementary Materials S1–S11. The analytical code (Python scripts for the random-effects meta-analysis, the multilevel meta-regression, and the leave-one-study-out sensitivity analyses) is available from the corresponding author on reasonable request.
